# Prostaglandin E_2_ Induces Long-Lasting Inhibition of Noradrenergic Neurons in the Locus Coeruleus and Moderates the Behavioral Response to Stressors

**DOI:** 10.1523/JNEUROSCI.0353-23.2023

**Published:** 2023-11-22

**Authors:** Yasutaka Mukai, Tatsuo S. Okubo, Michael Lazarus, Daisuke Ono, Kenji F. Tanaka, Akihiro Yamanaka

**Affiliations:** ^1^Department of Neuroscience II, Research Institute of Environmental Medicine, Nagoya University, Nagoya, Aichi 464-8601, Japan; ^2^Department of Neural Regulation, Nagoya University Graduate School of Medicine, Nagoya, Aichi 466-8550, Japan; ^3^Chinese Institute for Brain Research, Beijing 102206, China; ^4^International Institute for Integrative Sleep Medicine (WPI-IIIS) and Institute of Medicine, University of Tsukuba, Tsukuba, Ibaraki 305-8575, Japan; ^5^Division of Brain Sciences, Institute for Advanced Medical Research, Keio University School of Medicine, Shinjuku, Tokyo 160-8582, Japan; ^6^National Institute for Physiological Sciences, National Institutes of Natural Sciences, Okazaki, Aichi 444-8585, Japan

**Keywords:** calcium imaging, depression-like behavior, locus coeruleus, noradrenaline, prostaglandin E_2_, stress

## Abstract

Neuronal activity is modulated not only by inputs from other neurons but also by various factors, such as bioactive substances. Noradrenergic (NA) neurons in the locus coeruleus (LC-NA neurons) are involved in diverse physiological functions, including sleep/wakefulness and stress responses. Previous studies have identified various substances and receptors that modulate LC-NA neuronal activity through techniques including electrophysiology, calcium imaging, and single-cell RNA sequencing. However, many substances with unknown physiological significance have been overlooked. Here, we established an efficient screening method for identifying substances that modulate LC-NA neuronal activity through intracellular calcium ([Ca^2+^]_i_) imaging using brain slices. Using both sexes of mice, we screened 53 bioactive substances, and identified five novel substances: gastrin-releasing peptide, neuromedin U, and angiotensin II, which increase [Ca^2+^]_i_, and pancreatic polypeptide and prostaglandin D_2_, which decrease [Ca^2+^]_i_. Among them, neuromedin U induced the greatest response in female mice. In terms of the duration of [Ca^2+^]_i_ change, we focused on prostaglandin E_2_ (PGE_2_), since it induces a long-lasting decrease in [Ca^2+^]_i_ via the EP_3_ receptor. Conditional knock-out of the receptor in LC-NA neurons resulted in increased depression-like behavior, prolonged wakefulness in the dark period, and increased [Ca^2+^]_i_ after stress exposure. Our results demonstrate the effectiveness of our screening method for identifying substances that modulate a specific neuronal population in an unbiased manner and suggest that stress-induced prostaglandin E_2_ can suppress LC-NA neuronal activity to moderate the behavioral response to stressors. Our screening method will contribute to uncovering previously unknown physiological functions of uncharacterized bioactive substances in specific neuronal populations.

**SIGNIFICANCE STATEMENT** Bioactive substances modulate the activity of specific neuronal populations. However, since only a limited number of substances with predicted effects have been investigated, many substances that may modulate neuronal activity have gone unrecognized. Here, we established an unbiased method for identifying modulatory substances by measuring the intracellular calcium signal, which reflects neuronal activity. We examined noradrenergic (NA) neurons in the locus coeruleus (LC-NA neurons), which are involved in diverse physiological functions. We identified five novel substances that modulate LC-NA neuronal activity. We also found that stress-induced prostaglandin E_2_ (PGE_2_) may suppress LC-NA neuronal activity and influence behavioral outcomes. Our screening method will help uncover previously overlooked functions of bioactive substances and provide insight into unrecognized roles of specific neuronal populations.

## Introduction

Neuronal activity is modulated by various factors, such as the composition of membrane proteins, the ionic balance of intracellular and extracellular conditions, and the presence of receptor-binding bioactive substances. Among those factors, various bioactive substances play a substantial role in modulating neuronal activity. These substances generally bind to specific receptors on the cell membrane to induce the opening or closing of ion channels and/or the activity of downstream signal transduction cascades, which ultimately modulate membrane excitability. Traditionally, the effects of such substances on neuronal activity have been studied using electrophysiological recording (Neher and Sakmann, 1975; Aston-Jones et al., 1991) and calcium signal recording (Miledi et al., 1980; Murai et al., 1997) with application of substances. However, these studies often focused on just a few substances for which a physiological function was already expected. Therefore, many substances with little-known physiological importance have been overlooked. Nowadays, single-cell RNA sequencing (scRNAseq; Zeisel et al., 2015; Mulvey et al., 2018) provides a nearly comprehensive dataset of receptor expression in a specific subtype of neurons. Nevertheless, it does not provide information on how substances actually modulate the activity of the neurons, such as time scale, amplitude, and direction of activity change. To address these inadequacies, we have established an efficient screening method for identifying substances that modulate the activity of a specific neuronal population. In this study, we introduced intracellular calcium imaging in the brains of transgenic mice that innately express calcium indicators, as an alternative to our previous method that required a viral infection to express calcium indicators (Mukai et al., 2020).

Noradrenergic (NA) neurons in the locus coeruleus (LC-NA neurons) are involved in diverse physiological functions, such as sleep/wakefulness (Berridge et al., 2012) and stress responses (Valentino and Bockstaele, 2008). The afferent and efferent neuronal circuitry has been investigated extensively (Schwarz et al., 2015; Poe et al., 2020), and various substances have also been reported to modulate LC-NA neuronal activity (Olpe and Steinmann, 1991; Szabadi, 2013). Nonetheless, many substances have yet to be examined for their function in LC-NA neurons. Here, we screened multiple bioactive substances for modulative function and found five novel substances that modulate the activity of LC-NA neurons in mouse brain slices. In addition, prostaglandin E_2_ (PGE_2_) showed an intriguing, long-lasting suppressive effect. Therefore, we further investigated its physiological function in detail.

PGE_2_ is produced by various types of cells in the brain and is involved in stress-related behavioral modulation; there are four subtypes (EP_1_ to EP_4_) of PGE_2_ receptors (Furuyashiki and Narumiya, 2011). Among the subtypes, expression of the EP_3_ receptor (EP3) in LC-NA neurons was first reported in rats as mRNA (Ek et al., 2000). Recently, the function of EP3 was also reported as a consequence of scRNAseq in mice (Mulvey et al., 2018). However, those investigators focused on the sexual dimorphism of EP3 expression in LC-NA neurons, in which expression is higher in female mice, and they used an artificial EP3 agonist, sulprostone, in their electrophysiological and behavioral experiments. Therefore, it remains unclear whether and how the endogenous agonist PGE_2_ modulates the activity of LC-NA neurons. Here, we demonstrate the possibility of endogenous PGE_2_ involvement in the suppression of the activity of LC-NA neurons and its behavioral effect by conditional knock-out of EP3 in mice.

## Materials and Methods

### Animals

All experiments were conducted following the ARRIVE guidelines 2.0 (Percie du Sert et al., 2020) and the Nagoya University Regulations on Animal Care and Use in Research. All experiments were approved by the Institutional Animal Care and Use Committee of the Research Institute of Environmental Medicine, Nagoya University (approval numbers R210096 and R210729). All efforts were made to reduce the number of animals used and to minimize the pain and suffering of the animals. Both sexes of *TetO YC* mice (*Actb^tm2.1(tetO-YCnano50)Kftnk^*; Kanemaru et al., 2014) on a mixed background, *D*β*H-tTA* mice (*Tg(Dbh-tTA)Kftnk*; Moriya et al., 2019) on a mixed background, *EP3-flox* (*Ptger3^tm1Csml^*) mice (Lazarus et al., 2007) on a C57BL/6J background, and *NAT-Cre* (*Tg(Slc6a2-cre)FV319Gsat*) mice (Gong et al., 2007) on a C57BL/6J background were used. Animals were maintained on a 12/12 h light/dark cycle under *ad libitum* feeding and drinking conditions. Room temperature was maintained at 23 ± 2°C.

### Buffers

The following buffers were used in this study: PBS containing (in mm) 137 NaCl, 2.7 KCl, 8 Na_2_HPO_4_, and 1.5 KH_2_PO_4_; KCl-based pipette solution containing (in mm) 145 KCl, 1 MgCl_2_, 10 HEPES, 1.1 EGTA, 2 adenosine-5′-triphosphate magnesium salt, and 0.5 guanosine-5′-triphosphate disodium salt, 280–290 mOsm, pH 7.3 with KOH; cutting solution containing (in mm) 15 KCl, 3.3 MgCl_2_, 110 K-gluconate, 0.05 EGTA, 5 HEPES, 25 glucose, 26.2 NaHCO_3_, and 0.0015 (±)-3-(2-Carboxypiperazin-4-yl)propyl-1-phosphonic acid; and artificial CSF (aCSF) containing (in mm) 124 NaCl, three KCl, 2 MgCl_2_, 2 CaCl_2_, 1.23 NaH_2_PO_4_, 26 NaHCO_3_, and 25 glucose. The cutting solution and aCSF were bubbled with carbogen gas (O_2_, 95%; CO_2_, 5%).

### Plasmids

The plasmids *pAAV-CMV-FLEX-YC-Nano50-WPRE* and *pAAV-TetO-YC-Nano50-WPRE* were produced in-house with a *Yellow Camelon-Nano50/pcDNA3* plasmid, which was kindly provided by Dr. Nagai (Horikawa et al., 2010). The plasmid *pAAV-CMV-FLEX-G-CaMP6* was produced in-house with the plasmid *pN1-G-CaMP6* (RIKEN RDB14609; Ohkura et al., 2012). “G-CaMP6” was obtained from Dr. Nakai and is distinct from “GCaMP6s/m/f” obtained from Janelia Research Campus (Chen et al., 2013). The plasmid *pAAV-TetO(3G)-mCherry-2A-Cre-WPRE* was produced in-house with synthetic DNA. In this construct, Cre recombinase is co-expressed with and separated from mCherry by an intermediary 2A-peptide sequence. The plasmid *pHelper* was purchased from Agilent Technologies, and the plasmid *pAAV-RC* (serotype 9) was kindly provided by the University of Pennsylvania vector core.

### Adeno-associated virus (AAV)

AAVs were generated according to a protocol described elsewhere (Izawa et al., 2019; Mukai et al., 2020). Briefly, *pHelper*, *pAAV-RC* (serotype 9), and any of the *pAAV* plasmids were transfected into AAV-293 cells (Agilent Technologies) using the calcium phosphate method. Three days after transfection, cells were collected. For the production of AAV9-CMV-FLEX-YC-Nano50-WPRE (AAV-CMV-FLEX-YC; 1.0 × 10^13^ copies/ml), AAV9-TetO-YC-Nano50-WPRE (AAV-TetO-YC; 6.2 × 10^13^ copies/ml), and AAV9-CMV-FLEX-G-CaMP6 (AAV-CMV-FLEX-G-CaMP6; 2 × 10^13^ copies/ml), cells were suspended in PBS, and AAVs were purified by ultracentrifugation. The final virus solvent was a mixture of PBS and OptiPrep (Alere Technologies AS), where the ratio of the solution depended on the AAV titer. For the production of AAV9-TetO(3G)-mCherry-2A-Cre-WPRE (AAV-TetO-Cre; 1.20 × 10^13^ copies/ml), cells were suspended in HBSS (H8264, Sigma-Aldrich). After four freeze-thaw cycles to move the AAV outside the cells, the AAV-containing cell lysate was treated with 250 U/ml of Benzonase Nuclease (71205, Millipore) at 37°C for 30 min, and then centrifuged three times at 17,800 × *g* for 10 min at 4°C, with the supernatant after each centrifugation used for the next centrifugation. The final supernatant was then aliquoted and stored at −80°C.

### Stereotaxic injections

Mice were anesthetized with 1–2% isoflurane and immobilized on a stereotaxic frame. Their scalps were opened, and the skull above the injection site was drilled. A glass pipette (GC150-10; Harvard Apparatus) made with a puller (P-97, Sutter Instrument) was used for injection of AAV. In the bilateral LC (in mm, AP −5.4 from the bregma, ML 0.9 from the midline, DV −3.0 from the brain surface), 600 nl/site of AAV solution was injected by air pressure pulses regulated with a Pneumatic Picopump (World Precision Instruments) with a pulse generator (SEN-7103, Nihon Kohden). The injected animals were used for subsequent experiments conducted at least two weeks after injection for imaging and at least four weeks after injection for behavioral experiments.

### Fixed brain slices

Mice were deeply anesthetized with isoflurane and perfused with 25-ml chilled saline followed by 25-ml chilled 10% formalin. After decapitation, each skull was carefully removed, and the brains were placed in chilled 10% formalin for postfixation overnight. After postfixation, brains were placed into PBS containing 30% sucrose for cryoprotection for at least 48 h. After cryoprotection, brains were placed into O.C.T. compound (Sakura Finetek Japan) and frozen at −80°C for 20 min, then placed into a −20°C cryostat (CM3050 S; Leica Biosystems) for at least 1 h. Embedded brains were fixed on a stage using O.C.T. compound and sliced at a thickness of 40 μm. The slices obtained were stored in PBS containing 0.05% NaN_3_ (PBS-NaN_3_) at 4°C until the time of subsequent experiments.

### Immunohistochemistry

One brain slice of each series of four brain slices (every 160 µm) containing the LC was used for immunohistochemistry. Slices were briefly rinsed with PBS containing 1% bovine serum albumin (BSA; A7906-500G, Sigma) and 0.25% Triton X-100 (35501-15, Nacalai Tesque; PBS-BX), and washed with PBS-BX three times for 10 min each time. After washing, the slices were incubated in PBS-BX containing primary antibodies overnight. The following primary antibodies and dilutions were used: mouse anti-green fluorescent protein (GFP) antibody (1220461, Wako), 1:1000; chicken anti-GFP antibody (GFP-1010, Aveslabs), 1:1000; rabbit anti-tyrosine hydroxylase (TH) antibody (AB154, Chemicon), 1:1000; rabbit anti-DsRed antibody (632496, Takara), 1:1000; and mouse anti-TH antibody (MAB318, Millipore), 1:4000. After incubation, the slices were briefly rinsed and washed with PBS-BX three times for 10 min each time. After washing, the slices were incubated in PBS-BX containing secondary antibodies for 2 h. The following secondary antibodies were used, each at a 1:1000 dilution: donkey anti-mouse immunoglobulin G (IgG) conjugated with CF488 (20014, Biotium) and CF594 (20115, Biotium), donkey anti-chicken immunoglobulin Y (IgY) conjugated with CF488 (20079, Biotium), and donkey anti-rabbit IgG conjugated with CF488 (20015, Biotium) and CF594 (20152, Biotium). After incubation, the slices were briefly rinsed and incubated in PBS-BX containing 2 μm of DAPI (043-18804, Wako) for 10 min. After incubation, the slices were briefly rinsed and washed with PBS-BX three times for 10 min each time. After washing, the slices were mounted onto glass slides and encapsulated with PBS containing 50% glycerol, and the cover glasses were sealed with nail polish. The preparations were stored at 4°C until observation.

### Microscopy

Brain slice preparations were imaged using an epifluorescent microscope (BZ-X710, KEYENCE) and a confocal microscope (LSM710, Carl Zeiss) to examine the expression of *TetO YC* and AAV-derived transgenes. The exposure time with the BZ-X and the gain with the LSM were adjusted so as not to generate saturated pixels in the obtained images.

### Acute brain slices

Animals were anesthetized with isoflurane and decapitated. Brains were immediately removed and incubated in ice-cold cutting solution. The brains were sliced at a thickness of 250 μm using a vibratome (VT1200S, Leica). Slices were incubated in aCSF at 35°C for 1 h and then at room temperature for at least 1 h.

### Electrophysiological recordings

A glass pipette was made from a glass capillary (GC150-10, Harvard Apparatus) using a puller (P-1000, Sutter Instrument) to have a pipette resistance of 4–10 MΩ. KCl-based pipette solution and aCSF were used as the internal solution for whole-cell recording and loose-cell attached recording, respectively. For patch clamp recordings, an amplifier (Axopatch 200B, Molecular Devices) and a digitizer (Axon Digidata 1550A, Molecular Devices) were used. To examine the YC signal, after identifying a cell expressing YC, the cell was punctured with a glass pipette and maintained in a whole-cell current clamp mode. A negative current was injected to suppress spontaneous firing. When the resting membrane potential was stable for >30 s, the command current (100–500 pA, 5 ms) was injected with a specific frequency (1, 2, 5, and 10 Hz) for 10 s sequentially, with a gap of >2 min between each frequency. Data were acquired with software (Clampex 10.7, Molecular Devices). To examine the effect of PGE_2_, after identifying a cell expressing YC, the cell was attached to a glass pipette without puncturing, and maintained in a loose-cell attached mode. When the YC signal was nearly stable, 1 μm of PGE_2_ was applied for 2 min via perfusion. Firing was recorded for at least 2 h.

### Calcium imaging

A brain slice was placed in a chamber perfused with aCSF at 1.5 ml/min. The slice was anchored with a harp to avoid movement. A microscope (BX51WI, Olympus) was equipped with two objective lenses (20× and 40×), a filter cube with a dichroic mirror for CFP excitation, an optical splitter (W-VIEW GEMINI, Hamamatsu Photonics) with bandpass emitters and a dichroic mirror for YFP/CFP recording, an electron-multiplying charge-coupled device (EMCCD) camera (iXon Ultra 897 or iXon Ultra 888, Andor, Oxford Instruments) and a light source (Spectra X, Lumencor; Niji, Bluebox optics; or LED430L5, Thorlabs). For excitation, blue light (440 ± 20 nm, 50–210 μW/mm^2^, 100 ms) was applied. The fluorescent signals for CFP and YFP were observed and recorded with software (MetaFluor, Molecular Devices).

### Substance screening

The method of substance screening is described elsewhere (Mukai et al., 2020). Briefly, to monitor cell autonomous effects and suppress the effects of synaptic inputs from other neurons, the voltage-gated sodium channel blocker tetrodotoxin (1 μm) was added to the aCSF. For a single brain slice, 12 substances, at most, were screened sequentially. Each candidate substance was dissolved in aCSF. Then, the solution was applied for 2 min via perfusion. The time between applications was at least 5 min. When any change in the calcium signal was observed, the next substance was not applied until the signal returned to baseline and was stable for an additional 5 min. As controls for detecting baseline, increased and decreased calcium concentrations, aCSF, glutamate and GABA were applied, respectively. Each substance was examined at least four times in different orders and combinations in multiple slices from different animals.

### Behavioral experiments

#### Restraint stress (RS)

An animal was restrained for 30 min in a 50 ml tube with holes that allowed ventilation.

#### Tail suspension test (TST)

An animal was suspended by its tail at a height >50 cm with tape 15 cm long for 6 min. Behavior was recorded with a video camera (HDR-CX560V, Sony).

### Vigilance state recording

Electrodes for the electroencephalogram (EEG) were made of screws (400201000010002000, Tomimori) soldered to covered wires (361046MHW, Phoenix Wire) soldered to a pin connector (PH-2x40SG, Useconn Electronics). Electrodes for the electromyogram (EMG) were made of covered wires (AS633, Cooner Wire). Each end of the wire was stripped in 1 and 3 mm, and the 1 mm end was soldered to the same pin connector as that of the EEG. Mice were anesthetized with 1–2% isoflurane and immobilized on a stereotaxic frame. The scalp was opened at the midline from behind the eyes to the back near the trapezius muscle. A hole in the skull above the unilateral frontal cortex, the unilateral occipital cortex, and the center of the cerebellum was drilled at 0.7 mm in diameter. The EEG electrodes were screwed into the drilled holes. The EMG electrodes were inserted into the bilateral trapezius muscle and anchored with a 2-mm diameter sphere of glue (#30533, Aron Alpha Super Jelly, Konishi). The pin connector attached to the EEG and EMG electrodes was placed above the skull and cemented with the skull and screws in dental cement (Repairsin, GC). The animals were allowed to recover for at least 3 d and tethered to a recording cable for habitation for at least 7 d before recording. Signals of the EEG, EMG, and an infrared (IR) activity sensor, and video from an IR camera were recorded with a Vital Recorder (Kissei Comtec). The EEG and EMG signals were amplified and filtered (EEG: 1.5–30 Hz; EMG: 15–300 Hz) with an amplifier (AB-610J, Nihon Kohden).

### Fiber photometry surgery

Fiber cannula implantation was performed as described elsewhere (Mukai et al., 2023). Mice were anesthetized with 1–2% isoflurane and fixed on a stereotaxic frame. The scalp was opened at the midline from behind the eyes to the back of the head. A hole in the skull above the unilateral frontal cortex, the bilateral occipital cortex, the unilateral cerebellum, and above the LC (in mm, AP −5.6 from the bregma, ML 0.9 from the midline) was drilled at 0.7 mm in diameter. Anchors (400201000010002000, Tomimori) were screwed into the drilled holes. An optical fiber cannula with 400 µm in diameter, 6 mm length, and 0.39 NA, equipped with a ceramic ferrule of 1.25 mm in diameter and 6.4 mm in length (F0618S04B2P, Kyocera), was implanted above the LC (DV −2.9 mm from the brain surface). The fiber cannula and anchors were cemented with Super-Bond (C & B kit, Sun Medical) and further covered with dental cement (Repairsin, GC) containing bamboo charcoal powder (Taketora) to reduce light leakage.

### Fiber photometry recording

The recordings were performed with a custom apparatus. Products described in parentheses in this paragraph were all from Thorlabs unless otherwise specified. In this apparatus, the excitation of G-CaMP6 was achieved with a 470-nm LED (M470F3) through an excitation filter and a dichroic mirror involved in a filter set (MDF-GFP2) for a calcium-dependent signal and a 405-nm LED (M405FP1) through an excitation filter (FBH405-10) and a dichroic mirror (DMLP425R) for an isosbestic signal. Excitation light was transmitted to the implanted optical fiber cannula through optical fiber cables (M25L01), lenses (AC254-035-A-ML), irises (SM1D12C), filter cubes (DFM1/M), an objective lens (RMS20X-PF), and an optical fiber cable with 400 µm diameter and 0.39 NA (M95L01). The fluorescence emitted was transmitted to a photomultiplier tube (PMT; PMT1001/M) inversely through the optical fiber cable, the objective lens, the filter cube equipped with an emission filter in the filter set (MDF-GFP2), and a lens (AC254-040-A-ML). The intensities of the LEDs were controlled, and the PMT signal was collected by a multifunction I/O device (USB-6002, National Instruments) at a 1000 Hz time resolution controlled by a custom LabVIEW script (National Instruments). LEDs were illuminated alternately at 20 Hz with a duration of 25 ms. The intensities of the LEDs were adjusted to set the PMT signal around −1 and −0.5 V for 470- and 405-nm excitation-induced fluorescence, respectively.

### Code accessibility

Custom codes used in the current study are available from the corresponding authors on reasonable request.

### Experimental design and statistical analysis

Experiments were performed in individual animal cohorts for the following: brain slice imaging of screening and PGE_2_ concentration-response confirmation, receptor identification in cWT/cKO and icWT/icKO mice, TST in cWT/cKO and icWT/icKO mice, the vigilance state measurement in cWT/cKO mice, and fiber photometric measurement in cWT/cKO mice. Both sexes of animals were used. All statistical analyses were performed in OriginPro 2022 (Origin). All data are shown as the mean ± the standard error of the mean. The level of significance was set at *p* < 0.05. The number of regions of interest (ROIs) and the number and sex of animals for each analysis are provided in the figures and the tables. For calcium imaging data, the effective sample size (*n*_eff_) is also provided in the legends of the relevant figures and tables. Detailed procedures of the analyses in individual experiments are described below.

#### Image preparation for calcium imaging analysis

Calcium imaging analysis was performed as previously described (Mukai et al., 2020). Briefly, images of YFP and CFP were motion corrected and aligned using a custom MATLAB program based on the scale-invariant feature transform (Lowe, 2004). ROIs were drawn to surround cell bodies. ROIs were selected among cells that were present throughout the recording, and which were clearly distinguishable from other cells. ROIs that included two or more cells, or cells that disappeared before the end of an experiment, were omitted. YFP and CFP intensities were measured using Fiji software (Schindelin et al., 2012), and subsequent calculations that included the Y/C ratio were performed in MATLAB.

#### Calcium imaging analysis for electrophysiological confirmation of the YC signal

The raw Y/C ratio was used for analysis. The peak Y/C ratio during the current injection of each stimulation was subtracted from the mean Y/C ratio during the period 30 s before the current injection (R_0_) to obtain the peak ΔR. ΔR was divided by R_0_ to obtain the peak ΔR/R_0_.

#### Calcium imaging analysis for screening and confirmation of PGE_2_ concentration-response

The value of the Y/C ratio was corrected, and the Z-score of the corrected Y/C ratio was calculated for each session of substance, as described elsewhere (Mukai et al., 2020). The mean Z-score during the 5 min after the onset of each substance application was used. Quartiles of the mean Z-score for each substance were calculated from the combined screening experiments. When the third quartile was >2, the substance was defined as increasing [Ca^2+^]_i_; conversely, when the first quartile was less than −2, the substance was defined as decreasing [Ca^2+^]_i_. These definitions were established to enable conservative detection of a clear [Ca^2+^]_i_ change based on mean Z-scores observed after the application of glutamate, GABA and aCSF.

#### Calcium imaging analysis for receptor identification

The value of the Y/C ratio was corrected, and the Z-score of the corrected Y/C ratio was calculated for a session of PGE_2_, as described elsewhere (Mukai et al., 2020). The mean Z-score between 10 and 11 min after the onset of each substance application was used for comparison among wild-type, heterozygous, and homozygous animals of *EP3-flox*.

#### Full width at the half-maximum (FWHM) PGE_2_ signal

The Z-score of the corrected Y/C ratio (Z_YC_) was used for the analysis of the duration of signal change for each ROI and each concentration. ROIs whose mean Z-score during the 5 min after the onset of PGE_2_ was not less than −2 were excluded from the analysis at each concentration. The minimum value of the moving average of 120 frames of Z_YC_ (Z_min120_) was calculated. Then, the first time at which Z_YC_ < 0.5 × Z_min120_ (T_from_) and the last time at which Z_YC_ < 0.5 × Z_min120_ (T_to_) were calculated. The FWHM was calculated as T_to_ − T_from_.

#### The effective sample size (*n*_eff_)

Since calcium imaging data consist of multiple ROIs in each slice from different animals, the data obtained can be clustered depending on the individual animals. Therefore, the effective sample size (*n*_eff_), which is the theoretical sample size if there is no clustering, was calculated in R using the ICC package (version 2.4), consistent with Yu et al. (2022).

#### Linear mixed-effects (LME) model

To examine the correlation among ROIs collected from the same animal, random intercepts in a linear mixed-effects (LME) model (Lazic, 2010; Aarts et al., 2014; Yu et al., 2022) were incorporated into the statistical analysis of the calcium imaging data. The analysis was performed in R using the nlme package (version 3.1), consistent with Yu et al. (2022). “Fixed effects” and “random effects” were included, respectively, as follows: “sex” and “animal identity” for Figure 2*K* (NMU sex difference), “substance” and “animal identity” for Figure 2*L* (NPY/PYY/PP), “substance and sex” and “animal identity and ROI identity” for both Figure 3*F*,*G* (PGE_2_ sex difference and concentration dependence), “genotype and sex” and “animal identity” for Figure 4*H* (genotype and sex difference of cWT/cKO), and “genotype” and “animal identity” for Figure 4*O* (icWT/icKO). Using the functions in the nlme package, for ANOVA based on the LME model, a Wald *F* test was performed. In addition, *p* values for multiple comparisons were adjusted by Tukey's method if applicable and described as “MC adjusted” in the main text and legends, except for the comparison of the effect of PGE_2_ concentration between females and males in Table 2, where Bonferroni's method was used.

#### Immobility analysis for the TST

The video frames from the TST recording were separated into JPEG files using video editing software (Premiere Pro 2019–2022, Adobe), and the area of each animal's body was calculated using a custom MATLAB script. The continuous wavelet transform of the time series of the area was then calculated, and the specific time series (TS_WLT_), in which the effect of pendulous movement was subtracted out, reflecting the animal's struggling movement, was used for further analysis. The standard deviation of TS_WLT_ was calculated (σ_WLT_), and the time at which TS_WLT_ was below σ_WLT_/2 was defined as immobility. In the fiber photometry experiment, as fiber cables recorded in the video files often disrupted accurate measurements of body movement, immobility timing was manually corrected using the video editing software to omit frames in which an animal was moving but judged as immobile and to collect frames in which an animal was not moving but the fiber cable was moving and judged as mobile.

#### Vigilance state analysis

Animal vigilance states were identified in each 4-s epoch and classified into three states: wakefulness (W), rapid eye movement (REM) sleep (R), and non-REM (NR) sleep. The recorded signals of EEG, EMG and IR activity sensor, and the video of the IR camera were used for the analysis of the vigilance state with software (SleepSign, Kissei Comtec). The initial automatic screening was performed to identify the states as follows: a higher signal of the IR activity sensor was identified as W, a higher EMG signal was identified as W, a higher amplitude of the δ band (1.5–4 Hz) wave of the EEG was identified as NR, a higher ratio of the amplitude of the θ band (5–8 Hz) to that of the δ band wave (θ ratio) of the EEG was identified as R, and other conditions were identified as the previous state. After screening, manual scoring was performed by observing the EEG, EMG, and video.

#### Fiber photometry analysis

Raw PMT signal data contained 25 mutually consecutive time points of fluorescent signals excited at 470 and 405 nm (*F*_470RAW1–25_ and *F*_405RAW1–25_, respectively) at every 50 time points, where a time point corresponded to a duration of 1 ms. In each fluorescent signal (*F*_470RAW1–25_ and *F*_405RAW1–25_), the mean of signals between the 13th and 24th timepoints (*F*_470RAW13–24_ and *F*_405RAW13–24_) was used as a single data point (*F*_470@20Hz_ and *F*_405@20Hz_) corresponding to a duration of 25 ms. By following this procedure, we obtained a 20 Hz time series of *F*_470@20Hz_ and *F*_405@20Hz_. Then, the corrected time series of the G-CaMP6 signal (*F_GC_*) was calculated as *F*_470@20Hz_ − (*F*_405@20Hz_ × µ_470in1/3_/µ_405in1/3_), where µ_470in1/3_ and µ_405in1/3_ are the mean of the first third of the time series of *F*_470@20Hz_ and *F*_405@20Hz_, respectively. For the noise reduction, the moving average of 10 time points of *F*_GC_ during the TST (*F*_TS_) was calculated. Then, the relative G-CaMP6 signal during TS (*F*_TSre_) was calculated as (*F*_TS_ − *F*_TSmin_)/(*F*_TSmax_ − *F*_TSmin_), where *F*_TSmin_ and *F*_TSmin_ are the maximum and minimum values of *F*_TS_, respectively, as shown in Figure 6*E*,*F*. To further analyze the signal pattern, a set of the mobile-immobile period was defined as an epoch (Fig. 6*E*), and the maximum during the mobile (*F*_Emax_) and the minimum during the immobile period (*F*_Emin_) of each epoch were calculated. Then, the normalized G-CaMP6 signal during each epoch (*F*_Enorm_) was calculated as (*F*_E_ − *F*_Emin_)/(*F*_Emax_ − *F*_Emin_), where *F*_E_ is *F*_TSre_ of each epoch, as shown in Figure 6*G*,*H*. The value of *F*_Enorm_ at the onset of immobility (*F*_Eonset_) was calculated in each animal, and the mean value of *F*_Eonset_ in each animal was examined in Figure 6*I*.

## Results

### Screening of substances that regulate the activity of noradrenergic neurons in the locus coeruleus

**Figure 1.**
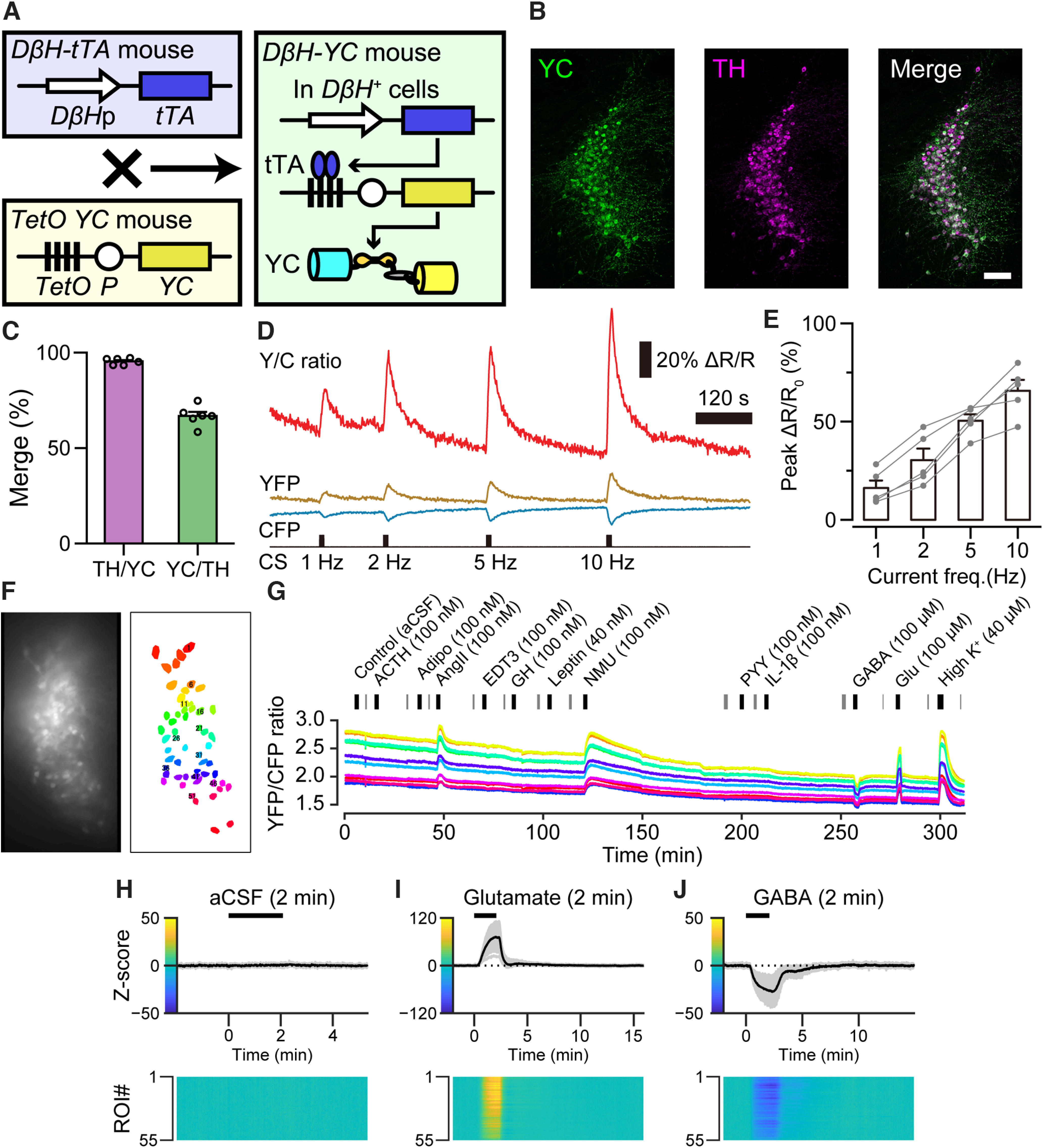
Screening of substances that regulate the activity of LC-NA neurons. ***A***, A schematic demonstrating the method of generation of the *D*β*H-YC* mouse strain. ***B***, Representative expression of YC in LC-NA neurons. Green, YC; Magenta, TH. Scale bar, 100 µm. ***C***, Results of cell counting. TH/YC, TH^+^ in YC^+^ indicating specificity, 95.4 ± 0.7%; YC/TH, YC^+^ in TH^+^ indicating efficiency, 66.8 ± 2.2%; *n* = 3 female and 3 male animals. ***D***, Representative trace of the change in YC signal induced by pulse current injections. YFP and CFP, fluorescence intensity (a.u.); Y/C ratio, YFP/CFP ratio calculated from YFP and CFP values; CS, current stimuli. The pulse frequency is shown at the bottom of the graph. ***E***, Peak ΔR/R_0_ value induced by pulse current injections. *n* = 5 cells from 1 female and 1 male animal. ***F***, Representative YC fluorescence signal (left) and ROIs (right). ***G***, Representative entire sequence of the Y/C ratio for one in every five ROIs (10 in total ROIs, shown as numbers in ***F***) of the brain shown in ***H–J***. The black bars indicate the timing of substance applications, and the gray bars indicate the timing of focus adjustments. ***H–J***, Z-scores of the Y/C ratio recorded from the brain slice shown in ***F***. The upper graphs show signal traces of individual ROIs (gray) and mean values (black). The black bars indicate the application timing (2 min) of each substance indicated above the graph (***H***, aCSF; ***I***, glutamate; ***J***, GABA). Heat maps show the Z-scores of individual ROIs indicated by the color bars at the *y*-axis of the upper graphs. *D*β*H*p, dopamine β-hydroxylase promoter; *tTA*, tetracycline trans-activator; *TetO*, tetracycline operator; *P*, minimal promoter; *YC*, yellow cameleon-Nano50; TH, tyrosine hydroxylase; aCSF, artificial CSF; GABA, γ-aminobutyric acid. Other abbreviations of substances in ***G*** are listed in Table 1.

To monitor the activity of LC-NA neurons, we generated a mouse strain expressing a calcium indicator, yellow Cameleon-Nano50 (YC) exclusively in NA neurons (*TetO YC;D*β*H-tTA*, YCD mice; Fig. 1*A*,*B*). We confirmed that YC was expressed in 66.8 ± 2.2% of LC-NA neurons at 95.4 ± 0.7% accuracy (*n* = 3 female and three male animals; Fig. 1*C*). Then, we examined the relationship between the YC signal and neuronal activity in an acute brain slice. We performed simultaneous calcium imaging and whole-cell patch clamp recording. YC is composed of yellow and cyan fluorescent proteins (YFP and CFP, respectively). When the intracellular calcium ion concentration ([Ca^2+^]_i_) is increased, the fluorescence of YFP and CFP will increase and decrease, respectively. Therefore, the ratio of YFP to CFP (Y/C ratio) is correlated with [Ca^2+^]_i_ (Kanemaru et al., 2014). Positive current pulse injection through the recording pipet induced an artificial action potential and increased the Y/C ratio in an applied pulse frequency-dependent manner (Fig. 1*D*,*E*). Thus, YC in LC-NA neurons in acute brain slices from YCD mice are functional for monitoring the activity of LC-NA neurons.

**Figure 2.**
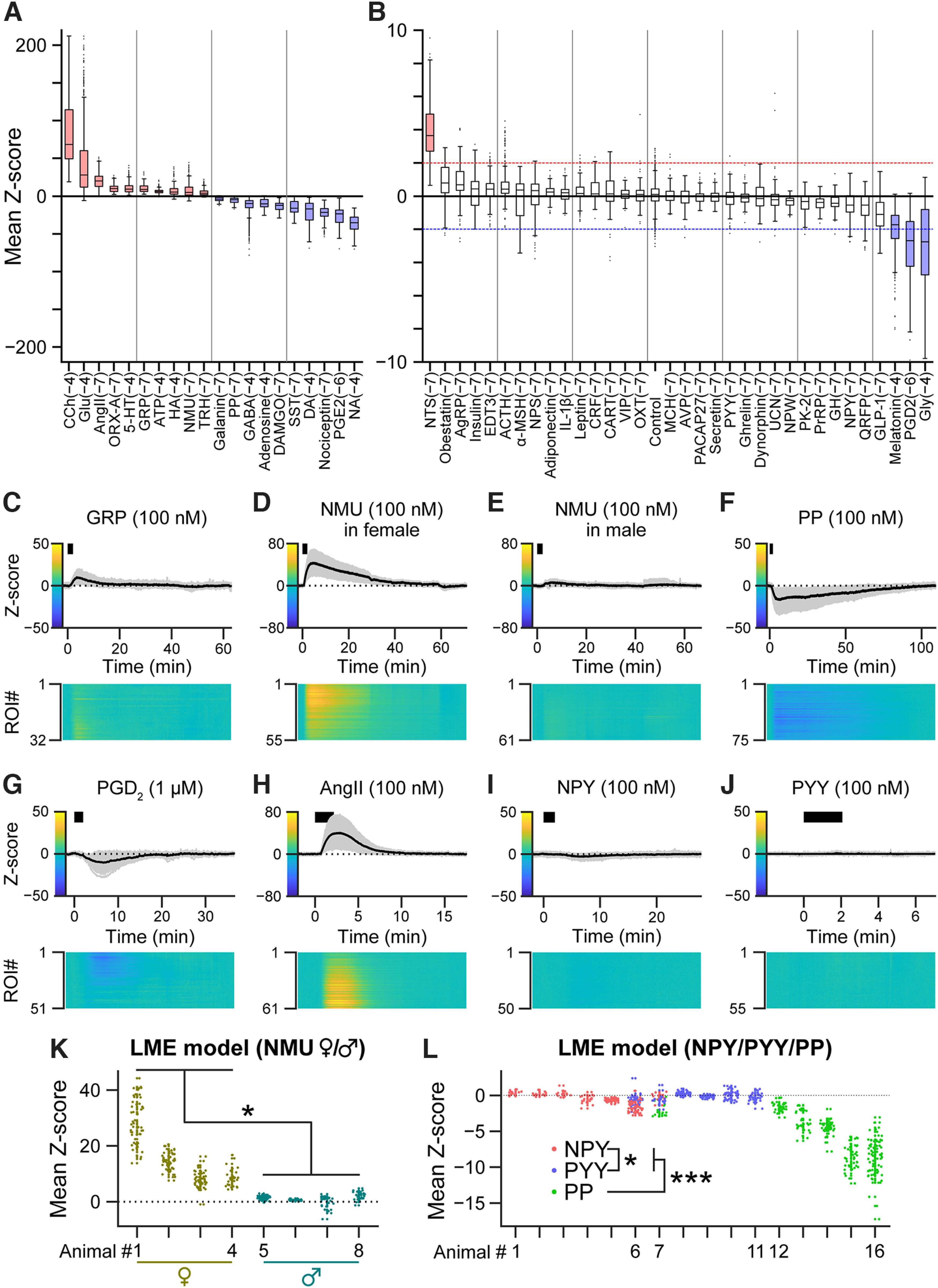
Identified substances that regulate the activity of LC-NA neurons. ***A***, ***B***, Box plots of the calcium signal changes induced by the substances indicated below. In the box plots, the top and bottom of each box indicate 75% and 25% points, respectively. The line inside the box indicates the median value. The upper and lower ends of the whiskers indicate the points not exceeding the interquartile range (IQR) × 1.5 from the edge of the box, where IQR = (the value of the 75% point) − (the value of the 25% point). Dots indicate outliers, which are data points beyond the whiskers. Substances are listed in the order of median value with the whiskers beyond 10 or −10 (***A***) and within 10 and −10 (***B***). Values in parentheses indicate the log_10_ concentration of the substances in mol/l. The red and blue dotted lines indicate where the mean Z-score = 2 and −2, respectively. Boxes filled in red or blue indicate the substances that increased or decreased intracellular calcium concentrations, respectively. *n* and abbreviations are listed in Table 1. ***C–J***, Z-scores of the Y/C ratio. The upper graphs show the signal traces of individual ROIs (gray) and mean values (black). The black bars indicate the application timing (2 min) of each substance indicated above the graph (***C***, GRP; ***D***, NMU in female; ***E***, NMU in male; ***F***, PP; ***G***, PGD_2_; ***H***, AngII; ***I***, NPY; ***J***, PYY). Heat maps show the Z-scores of individual ROIs indicated by the color bars at the *y*-axis of the upper graphs. ***K***, Individual plots of calcium signal changes induced by NMU in female and male animals. Data represent three female and three male animals from the screening cohort and one female (#2) and one male (#6) animal from an additional cohort. *n* = 187 ROIs from 4 female animals (the effective sample size, *n*_eff_ = 4.98) and 169 ROIs from 4 male animals (*n*_eff_ = 10.3); *p = 0.028, *t*_(6)_ = −2.88, based on the linear mixed-effects (LME) model. ***L***, Individual plots of calcium signal changes induced by NPY, PYY, and PP, in which data were reproduced from ***A*** and ***B*** for each substance. *n* = 173 ROIs from 7 animals (*n*_eff_ = 9.97; NPY), 252 ROIs from 6 animals (*n*_eff_ = 17.3; PYY), and 243 ROIs from 6 animals (*n*_eff_ = 7.84; PP); ***p < 0.001, *t*_(650)_ = 4.38 (NPY vs PP) and *t*_(650)_ = 5.96 (PYY vs PP), *p = 0.024, *t*_(650)_ = −2.62 (NPY vs PYY), based on the LME model, with multiple comparisons adjusted by Tukey's method. Note that animal # is not identical to ***K***. GRP, gastrin-releasing peptide; NMU, neuromedin U; PP, pancreatic polypeptide; PGD_2_, prostaglandin D_2_; AngII, angiotensin II; NPY, neuropeptide Y; PYY, peptide YY.

**Figure 3.**
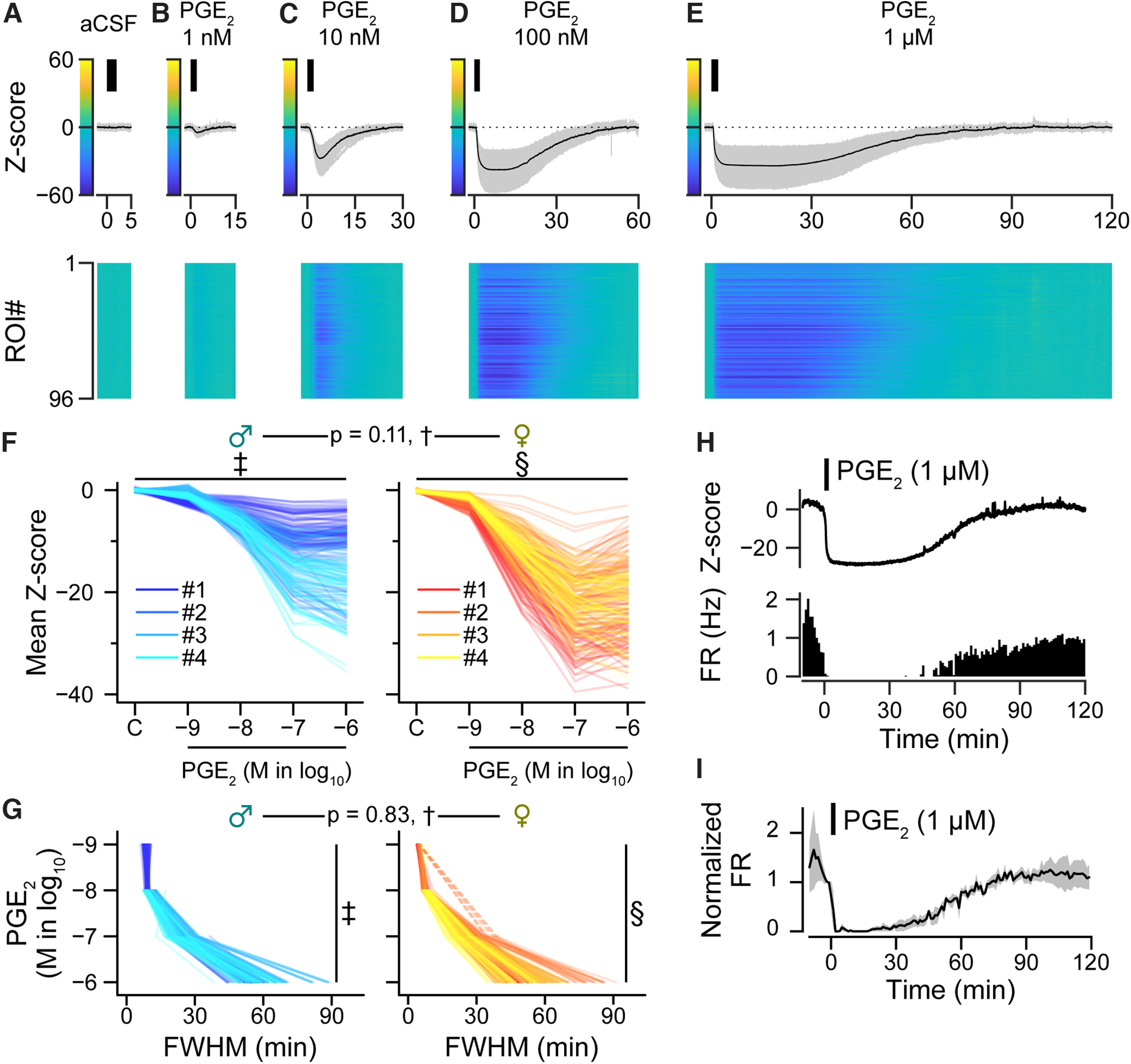
PGE_2_ suppressed the activity of LC-NA neurons in a concentration-dependent manner. ***A–E***, Representative Z-scores of the Y/C ratio. The upper graphs show signal traces of individual ROIs (gray) and mean values (black). The black bars indicate the application timing (2 min) of each substance indicated above the graph (***A***, aCSF; ***B–E***, PGE_2_ with different concentrations indicated above). Heat maps show the Z-scores of individual ROIs indicated by the color bars at the *y*-axis of the upper graphs. ***F***, Spaghetti plots of calcium signal changes (the mean Z-score during the 5 min after the onset of each substance application) in female and male animals induced by the substances indicated below, where C is aCSF, which was used as a control. Each line shows the value of an individual ROI. *n* = 239 and 237 ROIs from 4 male and 4 male animals; the effective sample size (*n*_eff_) = 46.7 and 16.2 (aCSF), 5.30 and 4.99 (1 nm), 6.71 and 7.23 (10 nm), 10.5 and 5.44 (100 nm), 7.04 and 5.08 (1 μm), female and male, respectively. †, ‡, and §, statistical values are listed in Table 2. ***G***, Spaghetti plots of the full width at the half-maximum (FWHM) Z-score of the Y/C ratio, which reflects the duration of calcium signal changes induced by the concentrations of PGE_2_ indicated on the *y*-axis. Each line shows the value of an individual ROI. Only ROIs that met the definition of “decrease” were used for the calculation: PGE_2_ 1 nm, *n* = 65 and 123 ROIs, *n*_eff_ = NaN and 4.06; 10 nm, *n* = 231 and 236 ROIs, *n*_eff_ = 5.60 and 19.5; 100 nm, *n* = 239 and 237 ROIs, *n*_eff_ =7.98 and 9.24; 1 μm, *n* = 238 and 237 ROIs, *n*_eff_ = 6.88 and 5.02; from 4 male and 4 female animals, respectively. The *n*_eff_ for males at 1 nm could not be calculated (NaN) because the FWHM was obtained from a single animal. Dotted lines in the female data show ROIs that met the definition of “decrease” at 1 nm, 10 nm, and 1 μm, but not at 10 nm PGE_2_. †, ‡, and §, statistical values are listed in Table 3. ***H***, Representative Z-score of the YFP/CFP ratio and firing rate (FR) of an identical neuron modulated by PGE_2_ application. ***I***, Normalized firing rate modulated by PGE_2_ application. *n* = 3 cells from 2 male animals.

Next, to screen for substances that regulate the activity of LC-NA neurons, we monitored [Ca^2+^]_i_ during and after the application of substances through perfused aCSF. To avoid the secondary effects of the input neurons, a voltage-gated sodium ion channel blocker, tetrodotoxin (TTX, 1 μm), was added to the perfused aCSF. As controls, aCSF itself, glutamate, and γ-aminobutyric acid (GABA) induced no change, an increase, and a decrease in the Z-scores of the Y/C ratio, respectively (Fig. 1*H–J*). Thus, our calcium imaging method can classify substances into those that do not change, increase, and decrease the activity of LC-NA neurons. Then, we applied candidate substances one by one through the perfused aCSF solution (Fig. 1*F*,*G*). We screened 53 bioactive substances, including five amines, three amino acids, one choline, two lipids, two nucleic acids, and 40 peptides (Table 1). We defined substances that increased or decreased the Y/C ratio from the Z-score, as described in Materials and Methods, to conservatively detect clear calcium changes based on the mean Z-scores observed after the application of glutamate, GABA, and aCSF.

**Table 1.** Screened substances

Substance	Abbreviation	Type	Slices	ROIs	*n*_eff_
Serotonin (5-hydroxytryptamine)	5-HT	Amine	f3/m5	f109/m76	10.7
Adrenocorticotropic hormone	ACTH	Peptide	f2/m2	f106/m128	6.03
Adenosine	Ade	Nuc. A.	f3/m3	f59/m124	7.90
Adiponectin	Adiponectin	Peptide	f2/m2	f95/m128	7.36
Agouti-related peptide	AgRP	Peptide	f2/m2	f57/m123	6.40
Angiotensin II	AngII	Peptide	f3/m3	f102/m143	10.5
Adenosine triphosphate	ATP	Nuc. A.	f3/m3	f68/m125	37.2
Arginine vasopressin	AVP	Peptide	f2/m4	f57/m64	16.1
Cocaine-and amphetamine-related transcript	CART	Peptide	f2/m2	f35/m73	24.0
Carbachol	CCh	Choline	f3/m3	f178/m126	8.87
Corticotropin releasing factor	CRF	Peptide	f2/m4	f75/m64	9.08
(Control)	(Control)	n.a.	f24/m22	f892/m829	65.4
Dopamine	DA	Amine	f3/m3	f122/m130	6.69
[d-Ala^2^, N-Me-Phe^4^, Gly^5^-ol]-enkephalin	DAMGO	Peptide	f5/m3	f103/m124	11.0
Dynorphin	Dyn	Peptide	f2/m2	f75/m70	6.90
Endothelin-3	EDT3	Peptide	f2/m2	f98/m128	4.78
γ-Aminobutyric acid	GABA	Ami. A.	f24/m22	f892/m829	53.5
Galanin	Galanin	Peptide	f3/m3	f69/m129	8.15
Growth hormone	GH	Peptide	f2/m2	f106/m128	7.60
Ghrelin	Ghrelin	Peptide	f3/m2	f69/m90	13.3
Glucagon-like peptide-1	GLP-1	Peptide	f3/m4	f129/m121	11.4
Glutamate	Glu	Ami. A.	f24/m22	f892/m829	59.8
Glycine	Gly	Ami. A.	f3/m3	f128/m73	8.41
Gastrin-releasing peptide	GRP	Peptide	f3/m3	f118/m67	10.6
Histamine	HA	Amine	f3/m3	f93/m116	9.04
Interleukin-1β	IL-1β	Peptide	f2/m2	f96/m103	5.87
Insulin	Insulin	Peptide	f3/m2	f127/m110	6.30
Leptin	Leptin	Peptide	f2/m2	f95/m128	7.81
Melanin-concentrating hormone	MCH	Peptide	f2/m4	f85/m64	24.5
Melatonin	MLTN	Amine	f3/m3	f111/m121	13.4
Noradrenaline	NA	Amine	f3/m3	f75/m98	11.4
Neurotensin	NTS	Peptide	f3/m3	f76/m125	14.3
Neuromedin U	NMU	Peptide	f3/m3	f136/m123	6.73
Nociceptin	Nociceptin	Peptide	f4/m3	f53/m109	9.15
Neuropeptide S	NPS	Peptide	f2/m4	f69/m64	14.6
Neuropeptide W	NPW	Peptide	f2/m2	f69/m35	10.1
Neuropeptide Y	NPY	Peptide	f3/m4	f109/m64	9.97
Obestatin	Obestatin	Peptide	f2/m2	f73/m117	4.27
Orexin-A	ORX-A	Peptide	f3/m3	f109/m124	8.66
Oxytocin	OXT	Peptide	f2/m5	f92/m76	22.5
Pituitary adenylate cyclase-activating polypeptide	PACAP27	Peptide	f2/m2	f84/m35	5.51
Prostaglandin D_2_	PGD2	Lipid	f3/m4	f69/m169	14.7
Prostaglandin E_2_	PGE2	Lipid	f5/m3	f77/m125	11.2
Prokineticin-2	PK2	Peptide	f2/m2	f75/m121	7.37
Pancreatic polypeptide	PP	Peptide	f3/m3	f102/m141	7.84
Prolactin-releasing peptide	PrRP	Peptide	f2/m2	f96/m121	7.45
Peptide tyrosine tyrosine	PYY	Peptide	f3/m3	f114/m138	17.3
Pyroglutamylated RF amide peptide	QRFP	Peptide	f2/m3	f93/m77	6.11
Secretin	Secretin	Peptide	f2/m2	f18/m94	7.36
Somatostatin	SST	Peptide	f3/m3	f112/m116	6.92
Thyrotropin-releasing hormone	TRH	Peptide	f3/m3	f122/m112	7.86
Urocortin-3	UCN	Peptide	f2/m2	f81/m35	51.0
Vasoactive intestinal peptide	VIP	Peptide	f2/m2	f61/m35	6.60
Alpha-melanocyte-stimulating hormone	α-MSH	Peptide	f2/m2	f35/m90	4.80

Substances used for screening are shown in the table listed by substance (1st column), abbreviation (2nd column), type (3rd column), *n* of slices (4th column) from female (f) and male (m) animals, *n* of ROIs (5th column) from female (f) and male (m) slices, and the effective sample size (*n*_eff_, 6th column). *Ami*. A., amino acid; *Nuc. A.*, nucleic acid; *n.a.*, not applicable.

The results showed that the Y/C ratio was increased by 11 substances: carbachol (CCh), glutamate (Glu), angiotensin II (AngII), orexin-A (ORX-A), serotonin (5-HT), gastrin-releasing peptide (GRP), adenosine triphosphate (ATP), histamine (HA), neuromedin U (NMU), thyrotropin-releasing hormone (TRH), and neurotensin (NTS; Fig. 2*A*,*B*). In contrast, the Y/C ratio was decreased by 13 substances: noradrenaline (NA), prostaglandin E_2_ (PGE_2_), nociceptin, dopamine (DA), somatostatin (SST), [d-Ala^2^, N-Me-Phe^4^, Gly^5^-ol]-enkephalin (DAMGO), adenosine, GABA, pancreatic polypeptide (PP), galanin, glycine (Gly), prostaglandin D2 (PGD_2_), and melatonin (Fig. 2*A*,*B*). Among the responsive substances, GRP (Fig. 2*C*), NMU (Fig. 2*D*,*E*), PP (Fig. 2*F*), and PGD_2_ (Fig. 2*G*) had not been reported in any preceding studies, and AngII (Fig. 2*H*) had not been reported in studies in mice. Interestingly, NMU increased the Y/C ratio significantly more in female animals than in male animals (*p* = 0.028, *t*_(6)_ = −2.88, based on the linear mixed-effects (LME) model; Fig. 2*D*,*E*,*K*), suggesting a sex difference. As a member of the neuropeptide Y (NPY) family containing NPY (Fig. 2*I*) and peptide YY (PYY; Fig. 2*J*), PP shares receptors with NPY and PYY (Pedragosa-Badia et al., 2013). PP showed a significantly lower value than NPY or PYY (*p* < 0.001, *t*_(650)_ = 4.38 (NPY vs PP) and 5.96 (PYY vs PP), based on the LME model, with multiple comparisons adjusted by Tukey's method (MC adjusted; Fig. 2*L*). There was also a significant difference between NPY and PYY (*p* = 0.024, *t*_(650)_ = −2.62; Fig. 2*L*). Upon further examination of the NPY signal, although a slight decrease in the Y/C ratio was observed around 5 min after application (Fig. 2*I*), it did not meet the criteria for a decrease in our screening method. In terms of the duration of [Ca^2+^]_i_ change, we also found that PGE_2_ induced a long-lasting decrease in the Y/C ratio (Fig. 3). Therefore, we further examined the effect of PGE_2_ on LC-NA neurons.

### PGE_2_ suppressed the activity of LC-NA neurons via EP3

To examine the concentration dependence of the PGE_2_ effect, we applied different concentrations of PGE_2_ (from 1 nm to 1 μm). Since a sex difference has previously been reported (Mulvey et al., 2018), we examined the effect separately in female and male animals (four female and four male animals). Results demonstrated that the depth and duration of the decrease in the Y/C ratio were positively correlated with PGE_2_ concentration in both female and male animals (Fig. 3*A–G*). In our experiment, there were no significant differences between female and male animals with regard to both overall calcium signal change depth and duration (depth, *p* = 0.11, *F*_(1,6)_ = 3.59, Fig. 3*F*; duration, *p* = 0.83, *F*_(1,6)_ = 0.0491, Fig. 3*G*; each based on the LME model, Wald *F* test). However, there were significant differences in the interaction between the factors of “sex” and “concentration” in both depth and duration (depth, *p* < 0.0001, *F*_(4,2364)_ = 153.76, Fig. 3*F*; duration, *p* < 0.0001, *F*_(3,1592)_ = 21.08, Fig. 3*G*; based on the LME model, Wald *F* test). *Post hoc* multiple comparison testing revealed that 100 nm of PGE_2_ induced significantly decreased [Ca^2+^]_i_ at deeper depths in females than in males (*p* = 0.025; Table 2), and higher concentrations of PGE_2_ significantly decreased [Ca^2+^]_i_ at deeper depths and for longer durations in both sexes (Table 2 and Fig. 3*F*; Table 3 and Fig. 3*G*). To examine whether the decrease in [Ca^2+^]_i_ reflects a decrease in membrane excitability, we simultaneously performed calcium imaging and loose-cell attached patch clamp recording without TTX. The application of PGE_2_ (1 μm) decreased the firing rate and the Y/C ratio (Fig. 3*H*,*I*). These results indicate that PGE_2_ suppresses the activity of LC-NA neurons.

**Table 2.** Statistical values from the analysis shown in Figure 3*F*

Combination	df	*t*	*p*
Control: female vs male	6	0.74	1.00
1 nm: female vs male	6	0.33	1.00
10 nm: female vs male	6	−2.99	0.12
100 nm: female vs male	6	−4.32	0.025
1 μm: female vs male	6	−3.11	0.10
Female: control vs 1 nm	2364	5.95	<0.0001
Female: 1 nm vs 10 nm	2364	30.9	<0.0001
Female: 10 nm vs 100 nm	2364	27.5	<0.0001
Female: 100 nm vs 1 μm	2364	3.27	0.0096
Male: control vs 1 nm	2364	3.72	0.0019
Male: 1 nm vs 10 nm	2364	30.9	<0.0001
Male: 10 nm vs 100 nm	2364	20.7	<0.0001
Male: 100 nm vs 1 μm	2364	3.34	0.0077

Calculated statistical values based on the linear mixed-effects (LME) model are shown in the table listed by combination of comparison (1st column), degree of freedom (df, 2nd column), *t* value (*t*, 3rd column), and *p* value (*p*, 4th column). Multiple comparisons were adjusted by Bonferroni's method for female versus male comparisons within each concentration and by Tukey's method for concentration comparisons in each sex. Concentrations are of PGE_2_.

**Table 3.** Statistical values from the analysis shown in Figure 3*G*

Combination	df	*t*	*p*
1 nm: female vs male	NaN	NaN	NaN
10 nm: female vs male	6	−0.134	1.00
100 nm: female vs male	6	0.591	1.00
1 μm: female vs male	6	−0.866	1.00
Female: 1 nm vs 10 nm	1592	−7.13	<0.0001
Female: 10 nm vs 100 nm	1592	−36.1	<0.0001
Female: 100 nm vs 1 μm	1592	−59.3	<0.0001
Male: 1 nm vs 10 nm	NaN	NaN	NaN
Male: 10 nm vs 100 nm	1592	−31.2	<0.0001
Male: 100 nm vs 1 μm	1592	−69.2	<0.0001

Calculated statistical values based on the linear mixed-effects (LME) model are shown in the table listed by combination of comparison (1st column), degree of freedom (df, 2nd column), *t* value (*t*, 3rd column), and *p* value (*p*, 4th column). Multiple comparisons were adjusted by Bonferroni's method for female versus male comparisons within each concentration and by Tukey's method for concentration comparisons in each sex. Concentrations are of PGE_2_. Some values could not be calculated (NaN) because the data for males at 1 nm was from a single animal.

**Figure 4.**
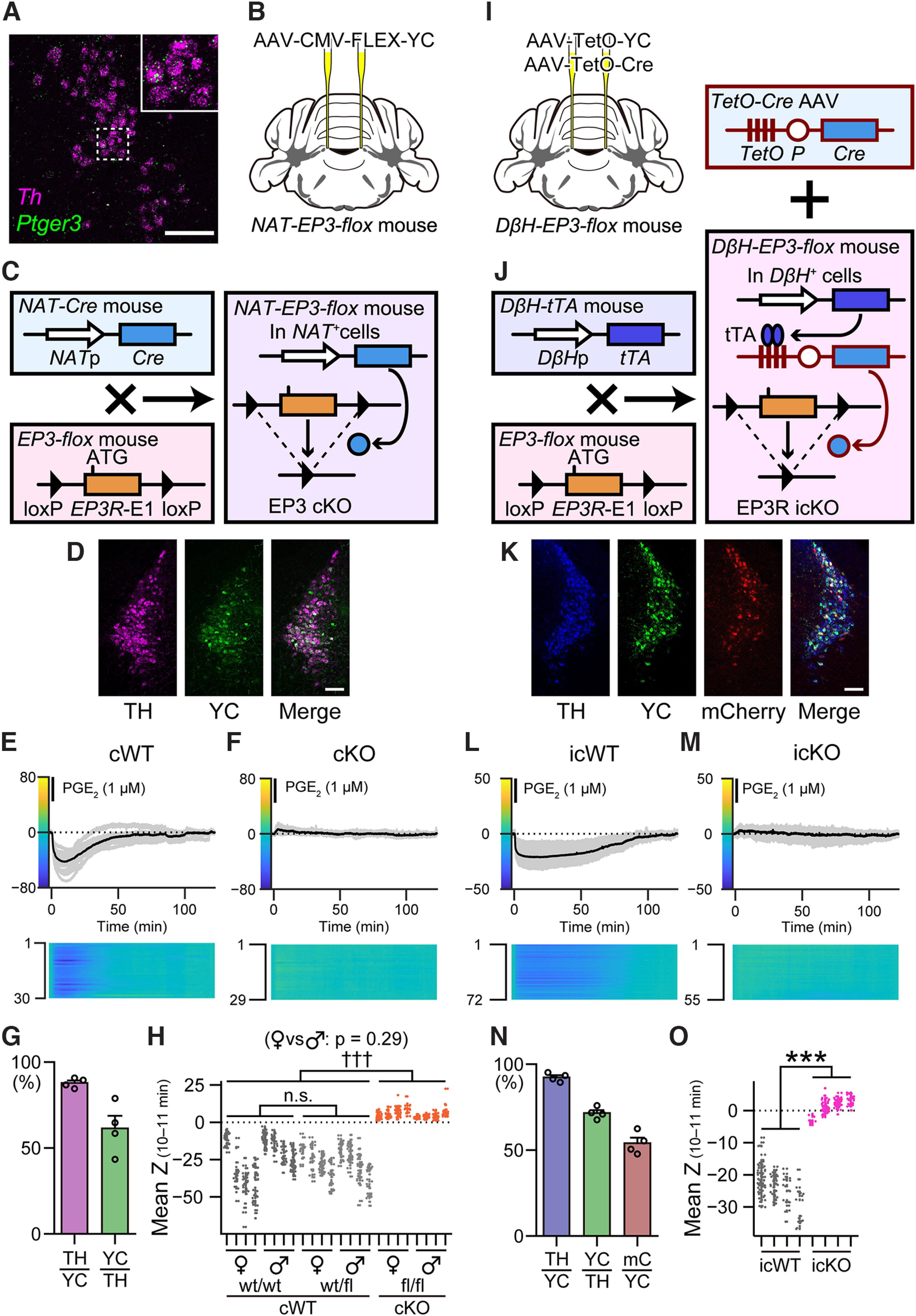
PGE_2_ suppressed the activity of LC-NA neurons via EP3. ***A***, Expression of EP3 mRNA (*Ptger3*, green) in LC-NA neurons expressing TH mRNA (*Th*, magenta). The inset shows a magnified image of a dotted square. Scale bar, 100 µm. ***B***, A schematic showing the location of AAV injection. ***C***, A schematic showing the generation of the *NAT-EP3-flox* mouse strain. ***D***, Representative expression of AAV-CMV-FLEX-YC in LC-NA neurons. Magenta, TH; Green, YC. Scale bar, 100 µm. ***E***, ***F***, Representative Z-scores of the YFP/CFP ratio recorded in cWT (***E***) and cKO (***F***) animals. ***G***, Results of cell counting. TH/YC, TH^+^ in YC^+^ indicating specificity, 87.9 ± 1.4%; YC/TH, YC^+^ in TH^+^ indicating efficiency, 61.3 ± 7.4%; *n* = 4 animals. ***H***, Individual plots of the mean of Z-scores of individual animals 10–11 min after the onset of PGE_2_ application (mean Z_(10–11 min)_). *n* = 4 each of female and male wt/wt, wt/fl, and fl/fl animals; ROIs and the effective sample size (*n*_eff_) = 119 and 5.56 (female wt/wt), 108 and 10.0 (female wt/fl), 110 and 7.00 (female fl/fl), 148 and 5.95 (male wt/wt), 149 and 13.7 (male wt/fl), 141 and 6.35 (male fl/fl); female vs male, *F*_(1,18)_ = 1.21, based on the linear mixed-effects (LME) model, Wald *F* test; †††*p* < 0.0001 (wt/wt vs fl/fl, *t*_(21)_ = 6.77; wt/fl vs fl/fl, *t*_(21)_ = 7.20), n.s. *p* = 0.90 (wt/wt vs wt/fl, *t*_(21)_ = 0.433), based on the LME model, with multiple comparisons adjusted by Tukey's method. ***I***, A schematic showing the location of AAV injection. ***J***, A schematic of the generation of the *D*β*H-EP3-flox* mouse strain. ***K***, Representative expression of AAV-TetO-YC and AAV-TetO-Cre in LC-NA neurons. AAV-TetO-Cre co-expressed mCherry (see Materials and Methods). Blue, TH; green, YC; red, mCherry. Scale bar, 100 µm. ***L***, ***M***, Representative Z-scores of the YFP/CFP ratio recorded in icWT (***L***) and icKO (***M***) animals. ***N***, Results of cell counting. TH/YC, TH^+^ in YC^+^ indicating specificity, 92.3 ± 1.2%; YC/TH, YC^+^ in TH^+^ indicating efficiency, 71.5 ± 1.7%; mC/YC, mCherrry^+^ in YC^+^, 54.0 ± 3.2%; *n* = 4 animals. ***O***, Individual plots of the mean of Z-scores of individual animals 10–11 min after the onset of PGE_2_ application (mean Z_(10–11 min)_). *n* = 4 icWT and 4 icKO animals; ROIs and *n*_eff_ = 149 and 10.1 (icWT), 160 and 6.29 (icKO); *t*_(6)_ = 11.2, ***p < 0.0001, based on the LME model. EP3, prostaglandin E_2_ EP_3_ receptor; *NAT*p, noradrenaline-transporter promoter; *Cre*, Cre recombinase; *EP3R*-E1, the first exon of the *Ptger3* gene; ATG, start codon; cKO, conditional knock-out; AAV, adeno-associated virus; CMV, cytomegalovirus promoter; FLEX, flip-excision switches; TH, tyrosine hydroxylase; YC, yellow cameleon-Nano50; cWT, wild-type control for cKO; PGE_2_, prostaglandin E_2_; *D*β*H*, dopamine β-hydroxylase; *tTA*, tetracycline trans-activator; *TetO*, tetracycline operator; *Cre*, Cre recombinase; *P*, minimal promoter; icWT, wild-type control for icKO; icKO, inducible conditional knock-out; mC, mCherry.

Next, we examined the receptors involved in the suppressive effect of PGE_2_ in LC-NA neurons. Among the four subtypes of PGE_2_ receptors, only EP3 is an inhibitory G_i_-coupled receptor. We confirmed that EP3 mRNA (*Ptger3*) was expressed in LC-NA neurons by *in situ* hybridization (Fig. 4*A*). Therefore, we examined whether EP3 was involved in the suppression of LC-NA neuronal activity. We crossed the *noradrenaline-transporter* (*NAT*)*-Cre* mouse strain (Gong et al., 2007), which expresses Cre recombinase in NA neurons, with the *EP3-flox* mouse strain, in which the first exon of the EP3 gene (*Ptger3*) is floxed (Lazarus et al., 2007) to conditionally knock out EP3 in NA neurons (*NAT-Cre; EP3^fl/fl^*, fl/fl, or “cKO mice”; Fig. 4*C*). We injected an AAV vector (AAV-CMV-FLEX-YC), which expresses YC in the presence of Cre recombinase (Mukai et al., 2020), to monitor the activity of LC-NA neurons during and after PGE_2_ application (Fig. 4*B*,*D*). We confirmed that YC was expressed in 61.3 ± 7.4% of LC-NA neurons at 87.9 ± 1.4% accuracy (*n* = 4 animals; Fig. 4*G*). In wild-type (*NAT-Cre; EP3^wt/wt^*, wt/wt) and heterozygous (*NAT-Cre; EP3^wt/fl^*, wt/fl) littermates of cKO mice, which are referred to as “cWT mice” in contrast to cKO mice, PGE_2_ (1 μm) decreased the Y/C ratio of LC-NA neurons in both female and male animals (Fig. 4*E*,*H*). However, in cKO mice, PGE_2_ did not decrease, but instead, slightly increased the Y/C ratio of LC-NA neurons (Fig. 4*F*,*H*). We performed statistical analysis based on the LME model, and found no significant difference between female and male animals (*p* = 0.29, *F*_(1,18)_ = 1.21, Wald *F* test). As there was no interaction between the factors of “sex” and “genotype” (*p* = 0.13, *F*_(2,18)_ = 2.25, Wald *F* test), we performed *post hoc* multiple comparison tests by combining both female and male data. The signal for cKO (fl/fl) animals was significantly higher than both wt/wt and wt/fl (*p* < 0.0001, *t*_(21)_ = 6.67 and *t*_(21)_ = 7.20, respectively; Fig. 4*H*). On the other hand, there was no significant difference between wild-type (wt/wt) and heterozygous (wt/fl) animals (*p* = 0.90, *t*_(21)_ = 0.433, MC adjusted; Fig. 4*H*). Therefore, we treated wt/wt and wt/fl animals as a single cluster termed “cWT” in the following experiments. These results suggest that the PGE_2_-induced decrease in [Ca^2+^]_i_ in LC-NA neurons is mediated by EP3.

In cKO mice, EP3 was knocked out not only in the LC but in all NA neurons. Therefore, we prepared another mouse strain to confirm the involvement of LC-specific EP3 in the PGE_2_ effect. We crossed the *dopamine* β*-hydroxylase* (*D*β*H*)*-tetracycline trans-activator* (*tTA*) mouse strain (Moriya et al., 2019), which expresses tTA in NA neurons, with the *EP3-flox* mouse strain to produce the *D*β*H-tTA;EP3^fl/fl^* mouse strain (*D*β*H-EP3-flox*). tTA induces gene expression under a tetracycline operator (TetO; Gossen and Bujard, 1992; Loew et al., 2010; Inutsuka et al., 2016). Therefore, we injected an AAV (AAV-TetO-Cre) to express Cre recombinase in LC-NA neurons (Fig. 4*I*,*J*). We named the AAV-TetO-Cre inducible conditional knock-out “icKO,” and also named the *D*β*H-tTA; EP3^fl/fl^* mice injected with AAV-TetO-Cre “icKO mice.” We also named the *D*β*H-tTA; EP3^wt/wt^* or *D*β*H-tTA; EP3^wt/fl^* mice injected with AAV-TetO-Cre “icWT mice” in contrast to icKO mice. We also injected an AAV (AAV-TetO-YC) to monitor the activity of LC-NA neurons during and after PGE_2_ application (Fig. 4*I*,*K*). We confirmed that YC was expressed in 71.5 ± 1.7% of LC-NA neurons at 92.3 ± 1.2% accuracy, while mCherry, which was co-expressed with Cre recombinase, was observed in 54.0 ± 3.2% of YC-expressing neurons (*n* = 4 animals; Fig. 4*N*). As a result, PGE_2_ decreased the Y/C ratio in icWT mice (Fig. 4*L*), whereas PGE_2_ did not change the Y/C ratio in icKO mice (*p* < 0.0001, *t*_(6)_ = 11.2, based on the LME model; *n* = 4 icWT and four icKO animals; Fig. 4*M*,*O*). These results suggest that the PGE_2_-induced decrease in [Ca^2+^]_i_ in LC-NA neurons is mediated by EP3 expressed in LC-NA neurons.

### EP3 in NA neurons is involved in stress-induced behavioral alterations

**Figure 5.**
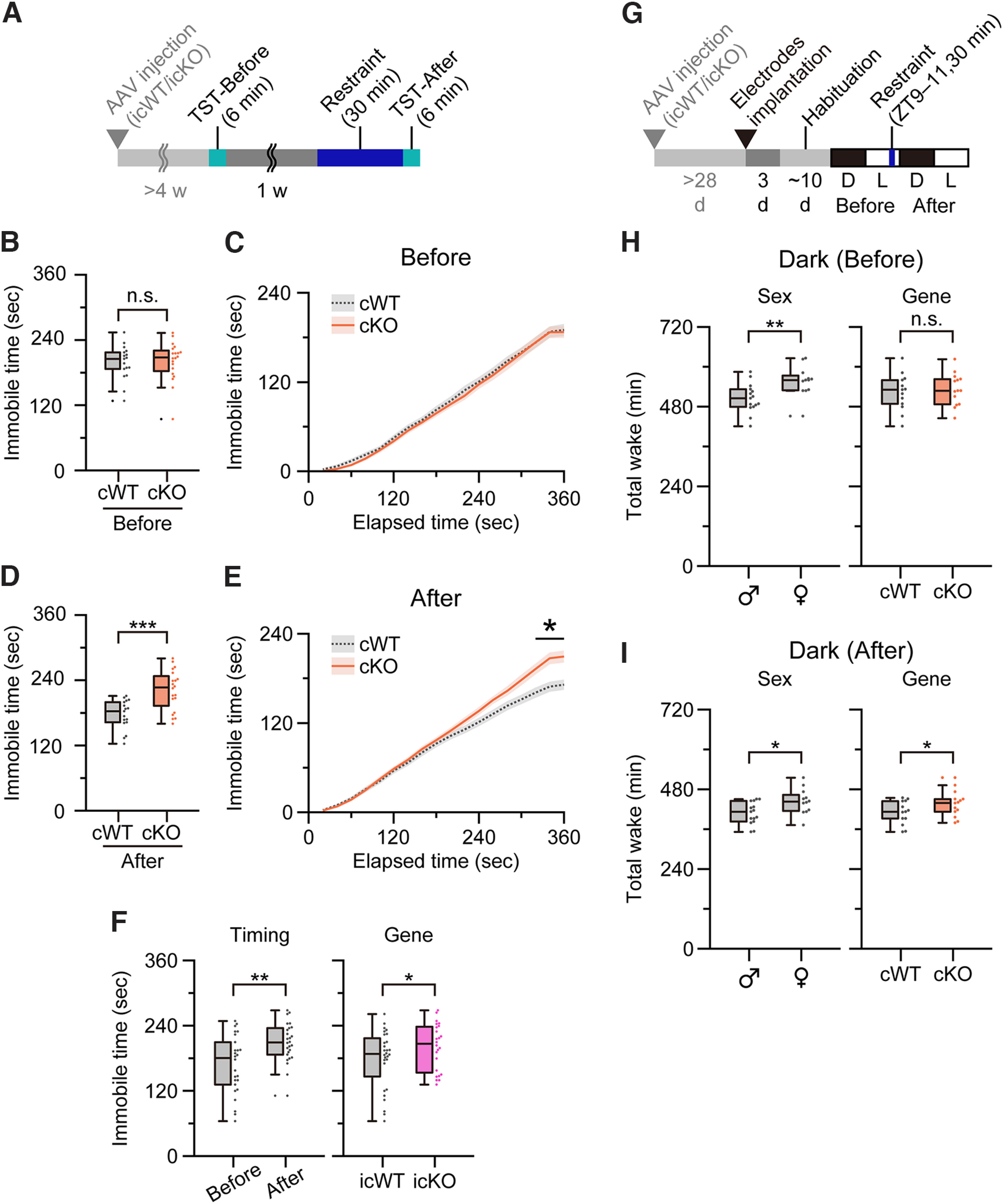
cKO animals showed increased depression-like behavior after restraint stress (RS). ***A***, A schematic of the tail suspension test (TST) before and after restraint stress. ***B***, Duration of immobility in cWT and cKO mice during the TST 1 w before RS. n.s., not significant, *p* = 0.97, *post hoc* Tukey's test after two-way repeated measures (RM) ANOVA. ***C***, Cumulative curve of duration of immobility shown in ***B*** across the TST 1 w before RS. ***D***, Duration of immobility in cWT and cKO mice during the TST after 30 min RS. ***p = 3.4 × 10^−4^, *post hoc* Tukey's test after two-way RM ANOVA. ***E***, Cumulative curve of the duration of immobility shown in ***D*** across the TST after 30-min RS. *p < 0.0024, Tukey's test (cWT vs cKO at 320, 340, and 360 s) after two-way RM ANOVA for cWT/cKO and 20-s binned elapsed time. In ***B–E***, *n* = 17 cWT and 20 cKO animals. ***F***, Duration of immobility in icWT and icKO mice during the TST before and after RS. Since two-way RM ANOVA showed an insignificant interaction (*p* = 0.22) between the factors of “Timing” (Before vs After) and “Gene” (icWT vs icKO), each factor was analyzed independently. *n* = 14 icWT and 12 icKO animals; **p = 0.0078 (Before vs After) and *p = 0.019 (icWT vs icKO), *post hoc* Tukey's test. ***G***, A schematic of sleep recording before and after RS. ***H***, ***I***, Total wake duration of cWT and cKO mice during the dark period before (***H***) and after (***I***) RS. Since two-way RM ANOVA showed an insignificant interaction in both cases (before, *p* = 0.89; after, *p* = 0.51) between the factors of “Sex” (male vs female) and “Gene” (cWT vs cKO), each factor was analyzed independently. **p = 0.0076 (male vs female) and n.s. *p* = 0.63 (cWT vs cKO), *post hoc* Tukey's test in ***H***. *p = 0.010 (male vs female) and *p = 0.041 (cWT vs cKO), *post hoc* Tukey's test in ***I***. *n* = 7 male cWT, 8 male cKO, 6 female cWT, and 6 female cKO animals. AAV, adeno-associated virus; d, day; w, week; TST, tail suspension test; cWT, wild-type control for cKO; cKO, conditional knock-out; icWT, wild-type control for icKO; icKO, inducible conditional knock-out.

Since PGE_2_ in the brain is involved in stress responses (Furuyashiki and Narumiya, 2011), we hypothesized that the activity of LC-NA neurons can be modulated by stress-induced PGE_2_ via EP3. Therefore, we compared the behavioral changes induced by restraint stressors that have been reported to modulate the activity of LC-NA neurons (McCall et al., 2015; Mulvey et al., 2018), in cKO and cWT animals. To assess depression-like behavior after RS, we performed the TST and measured the duration of the immobility (Can et al., 2011). We performed the TST without RS on day 1 (“Before”) and after RS on day 8 (“After”; Fig. 5*A*). The two-way repeated measures (RM) ANOVA showed a significant interaction (*p* = 0.0045) between the factors of “Timing” (Before vs After) and “Gene” (cWT vs cKO). Without RS on day 1 (“Before”), both cWT and cKO mice showed similar durations of immobility (*n* = 17 cWT and 20 cKO animals; *p* = 0.97, *post hoc* Tukey's test; Fig. 5*B*,*C*). In contrast, after RS (“After”), cKO mice showed longer durations of immobility than cWT animals (*p* = 3.4 × 10^−4^, *post hoc* Tukey's test; Fig. 5*D*,*E*). These results suggest that EP3 expressed in NA neurons is involved in the suppression of depression-like behavior after RS. To further confirm whether LC-specific EP3 is involved in this effect, we performed the TST in icWT and icKO animals. The two-way RM ANOVA showed no significant interaction between the factors of “Timing” (Before vs After) and “Gene” (icWT vs icKO; *p* = 0.22). Therefore, we compared each factor independently. We found that icKO mice (Fig. 5*F*, pink bar) showed significantly longer durations of immobility, while all animals showed longer durations of immobility after RS (*n* = 14 icWT and 12 icKO animals; *p* = 0.0078 for Before vs After and *p* = 0.019 for icWT vs icKO, *post hoc* Tukey's test; Fig. 5*F*). The difference in the significance of the interaction between cWT/cKO and icWT/icKO mice could be because of the difference in background strain (C57BL/6J and mixed strain, respectively). Nevertheless, the results suggest that EP3 expressed in LC-NA neurons is involved in the suppression of depression-like behavior, particularly after RS in the C57BL/6J strain.

**Figure 6.**
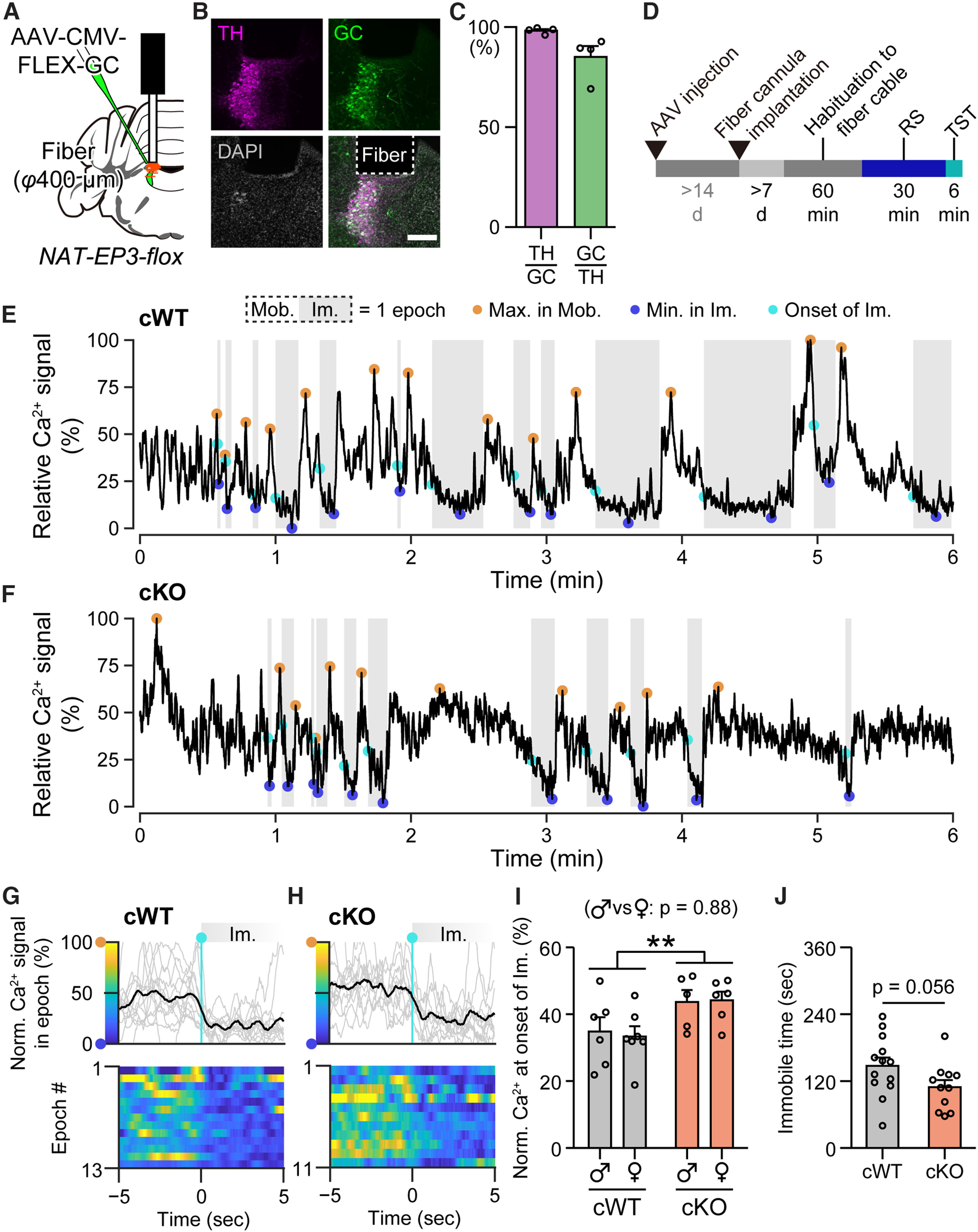
cKO animals showed higher LC-NA neural activity after restraint stress. ***A***, A schematic showing AAV injection and fiber cannula implantation. Red bars under the fiber cannula show the position of the tip of the fiber for the recorded animals (*n* = 24). ***B***, Representative expression of GC in LC-NA neurons, and location of the fiber above the LC-NA neurons. Magenta, TH; Green, GC. Scale bar, 200 µm. ***C***, Results of cell counting. TH/GC, TH^+^ in GC^+^ indicating specificity, 98.2 ± 0.8%; GC/TH, GC^+^ in TH^+^ indicating efficiency, 85.1 ± 5.4%; *n* = 4 animals. ***D***, A schematic showing AAV injection, fiber cannula implantation, and behavioral experiment. ***E***, ***F***, Representative calcium signal traces from cWT (***E***) and cKO (***F***) animals during the TST. White and gray backgrounds indicate the mobile (Mob.) and immobile (Im.) periods, respectively. A set of the mobile-immobile period was defined as an epoch. Orange, blue, and cyan circles indicate the maximum value in the mobile period, the minimum value in the immobile period, and the value at the onset of the immobile period, respectively. ***G***, ***H***, The normalized calcium signal in each epoch aligned to the onset of the immobile period in the cWT (***G***) and cKO (***H***) animals shown in ***E*** and ***F***, respectively. The upper graphs show the normalized calcium signal traces before and after 5 s from the individual onset of immobile periods. One hundred and 0% correspond to the maximum in the mobile period and the minimum in the immobile period of each epoch, respectively. Heat maps show the normalized calcium signals indicated by the color bars at the *y*-axis of the upper graphs. ***I***, The mean of the normalized calcium signals of each animal. Male cWT, 34.8 ± 4.3% (*n* = 6 animals); female cWT, 33.3 ± 3.1% (*n* = 7); male cKO, 43.7 ± 3.6% (*n* = 5); female cKO, 44.2 ± 2.6% (*n* = 6); **p = 0.0089, two-way ANOVA *post hoc* Tukey's test. ***J***, Duration of immobility during the TST. *p* value, Mann–Whitney *U* test. *n* = 13 cWT and 11 cKO animals. AAV, adeno-associated virus; CMV, cytomegalovirus promoter; FLEX, flip-excision cassette; GC, G-CaMP6; φ, diameter; TH, tyrosine hydroxylase; d, day; RS, restraint stress; TST, tail suspension test; cWT, wild-type control for cKO; cKO, conditional knock-out.

To further examine the difference between cKO and cWT animals at a longer period, which reflects the long-lasting characteristics of the PGE_2_ effect, we assessed sleep/wakefulness before and after RS. We implanted electrodes to record the electroencephalogram (EEG) and electromyogram (EMG) in cWT and cKO mice and recorded the EEG/EMG in isolated cages. After an animal was habituated in the cage for more than a week, we restrained the animal for 30 min within zeitgeber time (ZT) 9–11, and identified wakefulness, REM sleep, and non-REM sleep 24 h before and after RS (Fig. 5*G*). In the dark period before RS, although there was a significant sex difference (*p* = 0.0076, *post hoc* Tukey's test), cWT and cKO mice showed a comparable amount of total wakefulness (*p* = 0.63, *post hoc* Tukey's test; Fig. 5*H*). In contrast, in the dark period after RS, cKO animals had significantly longer wakefulness than cWT animals (*p* = 0.041, Tukey's test; Fig. 5*I*). It should be noted that, in cKO animals, since EP3 knock-out in NA neurons is not limited to the LC, behavioral outcomes can result, at least partially, from other NA neurons. These results suggest that EP3 in NA neurons is involved in stress-induced sleep/wake modulation.

### EP3 in NA neurons is involved in the modulation of the activity pattern of LC-NA neurons

Finally, we examined the activity of LC-NA neurons in cKO and cWT animals *in vivo*. To monitor the calcium activity in cKO and cWT animals, we injected an AAV (AAV-CMV-FLEX-G-CaMP6) that expresses a green-fluorescent calcium indicator, G-CaMP6 (GC; Ohkura et al., 2012), in a Cre-dependent manner. We implanted an optical fiber cannula above the unilateral LC (Fig. 6*A*,*B*). We confirmed that GC was expressed in 85.1 ± 5.4% of LC-NA neurons at 98.2 ± 0.8% accuracy (*n* = 4 animals; Fig. 6*C*). We performed fiber photometric recordings during the TST after RS (Fig. 6*D*). As a result, although an increase in calcium signal was synchronized with body movements during the TST (Fig. 6*E*,*F*), we found that the pattern of calcium activity was different between cKO and cWT mice; the timing of the decrease in calcium signal around the onset of immobility was delayed in cKO mice compared with cWT mice (*p* = 0.0089, two-way ANOVA *post hoc* Tukey's test; Fig. 6*G–I*), while there were no significant differences between females and males (*p* = 0.89, *F*_(1)_ = 0.020, two-way ANOVA) and no significant interaction between the factors of “sex” and “genotype” (*p* = 0.78, *F*_(1)_ = 0.082, two-way ANOVA). This result suggests that the activity of LC-NA neurons was less suppressed in cKO mice than in cWT mice, which was consistent with the suppressive function of G_i_-coupled EP3. With an optical fiber cable attached to the head, cKO mice tended to show a decrease in the duration of immobility compared with cWT mice (*p* = 0.056, Mann–Whitney *U* test; Fig. 6*J*), which was opposite of the experiment without fiber cables (Fig. 5*D*). This discrepancy may be because of the existence of a fiber cable on the head, which could provide more stimulation to the cKO mice to struggle.

## Discussion

In this study, we identified 24 candidate substances that modulate LC-NA neuronal activity in murine brain slices (Fig. 2*A*,*B*). Among them, GRP, NMU, and AngII increased [Ca^2+^]_i_, while PP and PGD_2_ decreased [Ca^2+^]_i_; these substances have not been previously reported to affect LC-NA neurons in mice (Fig. 2*C–H*). However, PGD_2_ may cross-affect EP3, causing decreases in [Ca^2+^]_i_ because of weak EP3 affinity (Boie et al., 1997).

GRP is a peptide involved in various physiological functions, such as sexual behavior (Sakamoto et al., 2008; Roesler and Schwartsmann, 2012). GRP belongs to the neuropeptide bombesin (BB) family, and mammalian receptors for BB family members include the neuromedin B (NMB) receptor, the GRP receptor (GRPR), and the bombesin receptor subtype 3. These receptors are known as G_q/11_-coupled GPCRs (Kroog et al., 1995; Jensen et al., 2008), and GRPR mediates an increase in [Ca^2+^]_i_ stimulated by GRP (Hellmich et al., 1999). Increased GRP immunoreactivity has been reported in the LC of suicide victims' postmortem brains (Merali et al., 2006). GRP-immunoreactive fibers have also been reported in the LC of cats (Marcos et al., 1994) and rainbow trout (Cuadrado et al., 1994). NMB is also a BB family neuropeptide (Kroog et al., 1995; Jensen et al., 2008), and radiolabeled NMB signal has been detected in the LC of rats (Lee et al., 1990). However, to the best of our knowledge, this is the first report demonstrating GRP/NMB activation of LC-NA neurons.

NMU is a peptide involved in various physiological functions, such as homeostatic regulation (Malendowicz and Rucinski, 2021). NMU shares target receptors with neuromedin S (NMS; Mori et al., 2005). NMU is expressed in multiple brain regions, including the mesencephalic trigeminal nucleus, which is adjacent to the LC (Honzawa et al., 1987), while NMS is specifically expressed in the suprachiasmatic nucleus (Mori et al., 2005). NMU/NMS have two known receptors: NMU receptor 1 (NMUR1) and NMU receptor 2 (Brighton et al., 2004a). Both receptors are GPCRs coupled with both G_q/11_ and G_i_, and show an increase in [Ca^2+^]_i_ on NMU application (Brighton et al., 2004b). *NMUR1* mRNA expression has been reported in the LC of humans (Szekeres et al., 2000). However, the function of NMU/NMS in LC-NA neurons remains unexplored. Interestingly, NMU induced a larger response in female mice than in males, suggesting potential sex-specific physiological functions.

PP is a peptide of the NPY family and is produced in the peripheral endocrine cells, particularly in the pancreas (Ekblad and Sundler, 2002), with potential brain access (Dumont et al., 2007). There are four known receptors for the NPY family, namely Y_1_, Y_2_, Y_4_, and Y_5_, and PP is highly selective for Y_4_ (Pedragosa-Badia et al., 2013). In the rodent LC, Y_1_, Y_2_, and Y_5_ mRNAs and proteins (Grove et al., 2000; Wolak et al., 2003; Theisen et al., 2018) and Y_4_ mRNAs (Parker and Herzog, 1999) are reported to be expressed. Various stressors modulate the expression of Y_1_, Y_2_, and Y_5_ mRNA in the LC of rats (Sabban et al., 2018; Bello et al., 2019; Nahvi et al., 2019; Nwokafor et al., 2019; Serova et al., 2019). Although the effects of NPY on LC-NA neurons have been studied (Finta et al., 1992; Illes et al., 1993; Kask et al., 1998; Lai and Lui, 2000), the effect of PP on LC-NA neurons remains unknown. All receptors of the NPY family are G_i/o_-coupled receptors (Cabrele and Beck-Sickinger, 2000) that can hyperpolarize the membrane potential (Lüscher and Slesinger, 2010), which may close voltage-gated Ca^2+^ channels and lead to a decrease in [Ca^2+^]_i_. Therefore, the decrease in [Ca^2+^]_i_ observed in the present study is consistent with the function of the NPY family of receptors. Remarkably, PP elicited a significantly larger effect compared with NPY and PYY (Fig. 2*F*,*I*,*J*), suggesting its potential physiological importance.

AngII, a peptide involved in fluid homeostasis (Paul et al., 2006), is produced and functions in the brain (Grobe et al., 2008; Matsuda et al., 2017) through AT_1_ and AT_2_ receptors (Bumpus et al., 1991). The existence of AngII receptors in the LC was suggested in rats (Mendelsohn et al., 1984). Expressed subtypes are controversial or different among species (Bregonzio et al., 2008), with predominant AT_2_ (Rowe et al., 1990; Song et al., 1991), exclusive AT_2_ (Tsutsumi and Saavedra, 1991), and dense AT_1_ (Phillips et al., 1993) reported in rats, while exclusive AT_1_ was reported in rabbits (Aldred et al., 1993) and mice (Häuser et al., 1998). Previous studies in rat brain slices showed that AngII did not affect NA efflux (Huang et al., 1987) and inhibited glutamate excitation via AT_2_ (Xiong and Marshall, 1990, 1994). However, there have been no reports in mice. The present study is the first to demonstrate that AngII increases [Ca^2+^]_i_ in LC-NA neurons in mice (Fig. 2*G*), consistent with the G_q_-coupled nature of the AT_1_ receptor (Tian et al., 1996).

We also examined the long-lasting suppressive effect of PGE_2_ in LC-NA neurons. Previously, EP_2_ and EP_4_ receptors (Zhang and Rivest, 1999), EP3 mRNA in the LC (Ek et al., 2000), and EP3 protein in LC-NA neurons (K. Nakamura et al., 2001) were reported in rats. Additionally, expression of EP3 mRNA in LC-NA neurons is higher in female than in male mice, and infusion of an EP3 agonist in the LC suppressed anxiety-like behavior in female but not in male mice (Mulvey et al., 2018). In the present study, we examined the effect of the endogenous EP3 agonist, PGE_2_, in LC-NA neurons of female and male mice. There was almost no significant difference in the depth and length of [Ca^2+^]_i_ decrease between female and male mice, except for 100 nm PGE_2_ to differ (*p* = 0.025; Fig. 3*F*,*G*; Table 2). Sex differences were also not significant in the calcium imaging of cWT/cKO animals (Fig. 4*H*). Sex differences may likely depend on the concentration of PGE_2_.

Furthermore, we introduced EP3-flox mice to conditionally knock out EP3 (cKO and icKO mice) and examined stress-induced behavioral modulation. In the TST, cKO animals showed longer durations of immobility, suggesting a more depression-like state than in cWT animals (Fig. 5*D*,*E*). The activity of LC-NA neurons is increased by various stressors (Valentino and Bockstaele, 2008; Zhai et al., 2023), and increased LC-NA neuronal activity can be correlated with depression-like states (Olson et al., 2011; Kurosawa et al., 2016; A. Nakamura et al., 2023). Therefore, in cKO animals, the lack of EP3 might have increased LC-NA neuronal activity after RS, causing greater depression-like behavior. In sleep recordings, cKO animals showed longer periods of wakefulness than cWT animals (Fig. 5*I*). Various stressors, including RS, induce longer sleep and thereby shorter wakefulness (Gonzalez et al., 1995; Pawlyk et al., 2008). Such stress-induced sleep may have an adaptive role for stressors (Feng et al., 2020). In cKO animals, lack of EP3 might have increased LC-NA neuronal activity after RS, facilitating the wake-promoting function of LC-NA neurons to increase wakefulness, even after RS. Additionally, LC-NA neuronal activity in cKO animals was higher than in cWT animals (Fig. 6*I*). PGE_2_ release in the brain is induced by stressors (Furuyashiki and Narumiya, 2011), while LC-NA neurons are activated by stressors (Valentino and Bockstaele, 2008). Therefore, stressor-induced PGE_2_ may suppress LC-NA neuronal activity through EP3 to moderate the animal's responses to stressors. RS is reported to increase the expression of PGE_2_-synthesizing enzymes in cortical neurons in rats (García-Bueno et al., 2008) and microglia in mice (Zhu et al., 2022).

In this study, we established an efficient method for screening substances that affect the activity of specific neurons using calcium imaging with endogenously YC-expressing transgenic mice with the KENGE-Tet system (Horikawa et al., 2010; Tanaka et al., 2012; Kanemaru et al., 2014). We previously reported a screening method using an AAV vector to express YC in Cre recombinase-expressing transgenic mice (Mukai et al., 2020), whereas the present study used endogenously YC-expressing transgenic mice. Researchers can choose between these two methods based on their specific research goals. To examine a specific subpopulation, one can use the AAV version, for example, by combination with retrograde AAV-Cre. To examine a general cellular population, one can use the transgenic mice version, for example, by crossing with a tTA-driver mouse strain (Ohmura et al., 2014; Tabuchi et al., 2014; Tsunematsu et al., 2014). It is important to note that there are several limitations of our screening method, which have been described in our previous report (Mukai et al., 2020). Recent omics studies, such as connectomics and transcriptomics, have powerfully revealed various characteristics of LC-NA neurons (Schwarz et al., 2015; Mulvey et al., 2018; Chandler et al., 2019). Nevertheless, our screening method identified five novel substances that modulate LC-NA neuronal activity in mice, demonstrating the efficiency and advantage of our screening method. This method will contribute to the identification of novel physiological functions of bioactive substances in specific neuronal populations.
